# Spatio-temporal activation of caspase-8 in myeloid cells upon ischemic stroke

**DOI:** 10.1186/s40478-016-0365-9

**Published:** 2016-08-26

**Authors:** Johanna Rodhe, Miguel A. Burguillos, Rocio M. de Pablos, Edel Kavanagh, Annette Persson, Elisabet Englund, Tomas Deierborg, Jose L. Venero, Bertrand Joseph

**Affiliations:** 1 Department of Oncology-Pathology, Cancer Centrum Karolinska, Karolinska Institutet, Stockholm, Sweden; 2 Departamento de Bioquímica y Biología Molecular, Universidad de Sevilla, and Instituto de Biomedicina de Sevilla-Hospital Universitario Virgen del Rocío/CSIC/Universidad de Sevilla, 41012 Sevilla, Spain; 3 Department of Clinical Sciences, Division of Oncology and Pathology, Department of Neuropathology, Lund University, Lund, Sweden; 4 Experimental Neuroinflammation Laboratory, Department of Experimental Medical Science, Lund University, Lund, Sweden

**Keywords:** Microglia, Macrophage, Caspase-8, Caspase-3, Ischemic stroke, Human brain tissue, pMCAO model, Spatio-temporal activation

## Abstract

Ischemic stroke (caused by thrombosis, embolism or vasoconstriction) lead to the recruitment and activation of immune cells including resident microglia and infiltrating peripheral macrophages, which contribute to an inflammatory response involved in regulation of the neuronal damage. We showed earlier that upon pro-inflammatory stimuli, the orderly activation of caspase-8 and caspase-3/7 regulates microglia activation through a protein kinase C-δ dependent pathway. Here, we present in vivo evidence for the activation of caspase-8 and caspase-3 in microglia/macrophages in post-mortem tissue from human ischemic stroke subjects. Indeed, CD68-positive microglia/macrophages in the ischemic peri-infarct area exhibited significant expression of the cleaved and active form of caspase-8 and caspase-3. The temporal and spatial activation of caspase-8 was further investigated in a permanent middle cerebral artery occlusion mouse model of ischemic stroke. Increasing levels of active caspase-8 was found in Iba1-positive cells over time in the peri-infarct area, at 6, 24 and 48 h after artery occlusion. Analysis of post-mortem brain tissue from human subject who suffered two stroke events, referred as recent and old stroke, revealed that expression of cleaved caspase-8 and -3 in CD68-positive cells could only be found in the recent stroke area. Analysis of cleaved caspase-8 and -3 expressions in a panel of human stroke cases arranged upon days-after stroke and age-matched controls suggested that the expression of these caspases correlated with the time of onset of stroke. Collectively, these data illustrate the temporal and spatial activation of caspase-8 and -3 in microglia/macrophages occurring upon ischemic stroke and suggest that the expression of these caspases could be used in neuropathological diagnostic work.

**Electronic supplementary material:**

The online version of this article (doi:10.1186/s40478-016-0365-9) contains supplementary material, which is available to authorized users.

## Introduction

Every year, an estimate of 15 million people worldwide suffers a stroke. As a result, nearly six million people die and an almost equal number of survivors are left with long-term disabilities [1, 2]. Stroke is an acute cerebrovascular accident, which occur due to deranged blood supply to the brain. There are two main types of stroke: ischemic, due to lack of blood flow, and hemorrhagic, due to bleeding. Ischemic stroke, which is caused by a vessel obstructive thrombosis, embolism or vasoconstriction, accounts for over 80 % of all incidents, is the focus of the present study [1–3].

A decrease or reduction in blood flow results in hypoxia and glucose deprivation, which can lead to neuronal damage and cell death. The center of the ischemic area, the ischemic core, is most affected by the reduction in blood flow and also suffers the more instant and severe damage of the tissue. The area surrounding the ischemic region, the penumbra, can receive low levels of blood flow from adjacent vascularized areas, resulting in slower development of neuronal damage. Injured and dying cells release damage-associated molecular patterns (DAMPs), which activate an immune response that is a major contributor to stroke pathophysiology. In fact, the immune response to acute cerebral ischemia triggers an inflammatory reaction that may last up to several months and plays a critical role in mediating post-ischemic damage of the tissue and secondary neurodegeneration in the penumbra [4].

The infiltration of blood-borne immune cells facilitated by disruption of the blood–brain barrier integrity following brain ischemic injury contributes to the neuroinflammation process. Nevertheless, the brain’s initial inflammatory response to ischemic event is primarily thought to be mediated by microglia, the brain resident immune cells. Microglia are highly dynamic cells, which constantly scavenge the brain for potential threats and can get rapidly activated upon detection of insults to the brain, danger-signals or changes in the brain microenvironment [5]. In response to the tissue damage, microglia become activated and migrate to the ischemic area. Microglia are a predominant source of proinflammatory mediators including cytokines (e.g. tumor necrosis factor and interleukin-1β), complement factors, free radicals, nitric oxide (NO), chemokines (e.g. CCL2 and CCL3) and prostaglandins, all of which potentially contribute to further neuronal dysfunction and death [6, 7].

Suppression of neuroinflammation using a variety of drugs were proven to be successful in reducing infarct volume and improving outcomes in experimental models of stroke [8]. Despite these promising preclinical trials, up to date, clinical trials using anti-inflammatory agents have failed to improve clinical outcomes [9]. Therefore, in order to revitalize interest for the therapeutic targeting inflammatory pathways for the treatment of acute ischemic stroke there is a need for comprehensive understanding of the time-dependent recruitment and activation of inflammatory immune cells.

Caspases, a family of cysteinyl aspartate-specific proteases, are best known as executioners of apoptotic cell death and their activation are considered as a commitment to cell death. However, caspases also function as regulatory molecules for immunity, cell differentiation and cell-fate determination [10]. We previously reported the existence of a caspase-dependent signalling pathway controlling microglia pro-inflammatory activation and associated neurotoxicity. We showed that the orderly activation of caspases -8 and -3/7, commonly known to have executioner roles in apoptosis, can promote pro-inflammatory activation of microglia in the absence of cell death [11]. Additionally, we recently obtained evidence that the activation of human monocytes/macrophages in response to a pro-inflammatory stimulus also rely on caspase-8 function [12]. In addition, increasing evidence strongly suggests that caspase-8 may play a critical role in IL-1β processing, with special relevance to the NLRP3 inflammasome activation [13]. NLRP3 inflammasome activation usually requires two signals or steps; signal 1 (priming) leading to NF-kB activation and signal 2 (typically ATP or nigericin) leading to NLRP3 machinery assembly (including at least NLRP3, the ASC (PYCARD) adaptor, and caspase-1) [14]. As previously stated, we first demonstrated the involvement of caspase-8 in NF-kB activation (priming) in microglia in response to diverse inflammatory stimuli [11], recently validated using conditional caspase-8 knockout mice specifically in the myeloid system [15]. The activation of NF-kB is critical for upregulating the transcription of both pro-IL-1β and NLRP3, as both are further needed for inflammasome formation and activation. A role of caspase-8 in mediating priming and activation of the NLRP3 inflammasome has also been demonstrated in primary macrophages [16]. Coimmunoprecipitation and confocal studies have demonstrated that caspase-8 is present in the NLRP3 inflammasome complex, where it is believed to be involved in cleavage and processing of procaspase-1 [13]. It is important to note that IL-1β activates T-cell-mediated innate immunity and promotes secondary ischemic damage during the subacute phase of ischemic brain injury [17]. Given the high number of danger-associated molecular patterns (DAMPs) released as a consequence of the ischemic damage, and the key roles of caspases in regulating brain immune functions, it is important to discern between the apoptotic and non-apoptotic roles of caspases upon ischemic stroke.

In the present study, we investigated in vivo and in post-mortem tissue from ischemic stroke subjects whether caspase-8 and caspase-3 activation, key players of the caspase-dependent signaling pathways regulating microglia and macrophages (MMs) pro-inflammatory activation, exhibited spatiotemporal features upon ischemic stroke.

## Materials and methods

### Human brain tissue

Human brain tissue from stroke subjects and controls were used in this study as approved by the Regional Ethical Review Board in Lund, Sweden (Dnr 2010-196). Stroke tissue from regions within the white matter of the frontal and parietal lobe was examined and compared to area- and age-matched controls. Both female and male cases were included in the study with an average age of 75 ± 9 years for stroke cases (*n* = 9) and 75 ± 11 years for controls (*n* = 5). Deceased individuals referred for autopsy were examined for stroke or/and diffuse ischemic damage in frontoparietal white matter, judged as relevant for particular ischemia susceptibility in the upper border zone areas. Three of these patients died from a clinically suspected or also radiologically ascertained stroke within the last 24 h. In two other patients, an older stroke was known and also detected radiologically in the basal ganglia and the thalamus, however not reported in the white matter, from where the tissue was macroscopically identified and sampled. In four cases found to have ischemic white matter damage, the autopsy referrals focused on cardiopulmonary disease, including myocardial infarcts in some cases. In the latter individuals, macroscopic examination of the cut brain revealed either clear-cut or suspected infarcts, which were later microscopically verified. For sampling from focal stroke, an area of approximately 10 mm^2^ and 5 mm thickness in the immediate vicinity/border of the lesion was taken for analysis. For diffuse ischemic damage (non-focal lesion, no complete tissue loss) a similar amount of tissue was sampled in the most damaged area.

The tissue was fixed in 4 % formaldehyde solution and embedded in paraffin, in accordance with the standard procedures within the department. Sections of 5 μm thickness were cut with microtome and mounted on glass slides. Tissue sections on regular slides were processed for standard/basic stainings (hematoxylin-eosin (HE) and luxol fast blue/cresyl violet (LFB) for myelin and cell structures. Tissue sections on positively charged glass slides were processed for immunohistochemistry. All brain sections were analyzed under the microscope for clinical diagnostic purpose before inclusion in the study.

### Animals and surgery

C57BL/6 mice used in this study were obtained from the Center of Production and Animal Experimentation (Espartinas, Spain). Experiments were performed at the University of Seville (Spain) in accordance with the Guidelines of the European Union Council (86/609/EU), following Spanish regulation for the use of laboratory animals approved by the Scientific Committee of the University of Seville. Animals were kept at diurnal conditions with ad libitum access to food and water. Experiments were conducted using 3-month-old male mice (*n* = 13). Electrocoagulation was used to induce a permanent middle cerebral artery occlusion (pMCAO), as described elsewhere [18]. Six to 48 h post pMCAO, animals were perfused with 4 % paraformaldehyde, pH 7.4, during deep isoflurane anesthesia and brains were removed and cryoprotected in sucrose. After freezing in isopentane at −40 °C, brains were cut in 25 μm thick coronal sections with a cryostat and mounted onto gelatin coated slides.

### Immunohistochemistry

Deparaffination of the human tissue sections was done using standard procedures in the department of Neuropathology. The sections were boiled during 15 min in 10 mM Citrate buffer pH 6.0 for antigen retrieval. Stainings were done using an automated immunostainer (TechMateTM 500 Plus, DAKO) with DAKO ChemMate Kit Peroxidase/3-3’diaminobenzidine.

Mouse tissue sections were washed and incubated for 20 min in 0.3 % hydrogen peroxide in methanol. After washes, the sections were incubated in blocking buffer containing 1 % goat serum (Vector) in TBS for 1 h. Incubation with primary antibodies diluted in 1 % goat serum and 0.25 % Triton-X 100 in TBS for 24 h. 3 × 10 min TBS washes was followed by incubation with biotinylated secondary antibodies diluted in 0.25 % Triton-X 100 in TBS for 2 h. Sections were thereafter incubated with ExtrAvidin®-Peroxidase solution (Sigma) and visualized with a standard diaminobenzidine/hydrogen peroxidase reaction for 5 min.

### Immunofluorescence

Deparaffination of the human tissue was done using 3 × 5 min incubations in Xylene, followed by 2 × 10 min in 100 % ethanol, 2 × 10 min in 95 % ethanol and 3 × 5 min in H_2_O.

Antigen retrieval was done for all tissue used for immunofluorescent staining by boiling in 10 mM Citrate buffer pH 6.0 for 10 min at 95 °C. When sections reached RT they were washed in TBS, before blocking in 5 % goat serum and 0.1 % Triton-X100 in TBS for 1 h. Incubation of primary antibody diluted in blocking buffer was done overnight at 4 °C. Washes in 3 × 10 min TBS was followed by 1 h by appropriate secondary antibody in 0.1 % Triton-X100 in TBS in RT. Sections were washed in TBS before 15 min incubation in 1 μg/ml Hoechst 33342 in RT, and followed by TBS wash. Autofluorescence removal reagent (#2160 Millipore) was used for the human tissue according to manufacturer’s recommendations.

### Antibodies

Primary antibodies detecting anti-human cleaved caspase-8 (Asp391) (18C8; #9496), anti-mouse cleaved caspase-8 (Asp387) (D5B2; #8592), anti-human and -mouse cleaved caspase-3 (Asp175) (5A1E; #9664), anti-human cleaved PARP (D64E10; #5625) were purchased from Cell Signaling Technology®, anti-human CD68 (PG-M1; #M0876) from Dako and anti-mouse Iba-1 (#NB100-1028) from Novus Biologicals and (#019-19741) from Wako. For immunofluorescence detection, fluorophore conjugated secondary antibodies were purchased from Invitrogen, ThermoFisher Scientific. Goat anti-rabbit Alexa Fluor®488 and goat anti-mouse Alexa Fluor®594 were used for the human tissue analysis, whereas donkey anti-rabbit Alexa Fluor®488 and donkey anti-goat Alexa Fluor®594 were used for mouse tissue analysis. Biotinylated goat anti-rabbit IgG were obtained from Vector Laboratories.

### Tissue analysis

Analysis of human tissue by Immunohistochemistry was done by a neuropathologist with a light microscope and scored for presence of cleaved caspases and upregulation of CD68 in the peri-infarct and ischemic area. Presence of cleaved caspase-8 and -3 as well as increased CD68 was semiquantitative assessed based on staining intensity of each antibody in both peak foci and the entire sampled region. The scoring was done blinded to other clinical information. Additional hematoxylin & eosin (HE) and luxol fast blue/cresyl violet (LFB) staining of all tissue was used to identify the stroke area and to evaluate the age of the ischemic lesions. The same areas were examined for the double immunofluorescent labelling of cells by Zeiss LSM510 and Zeiss LMS700 confocal laser scanning microscopy equipped with inverted Zeiss Axiovert 200 m microscopes using Zeiss LSM 5 and Zeiss ZEN 7.1 software.

### Immunohistochemistry cell quantification

**Fig. 1:**
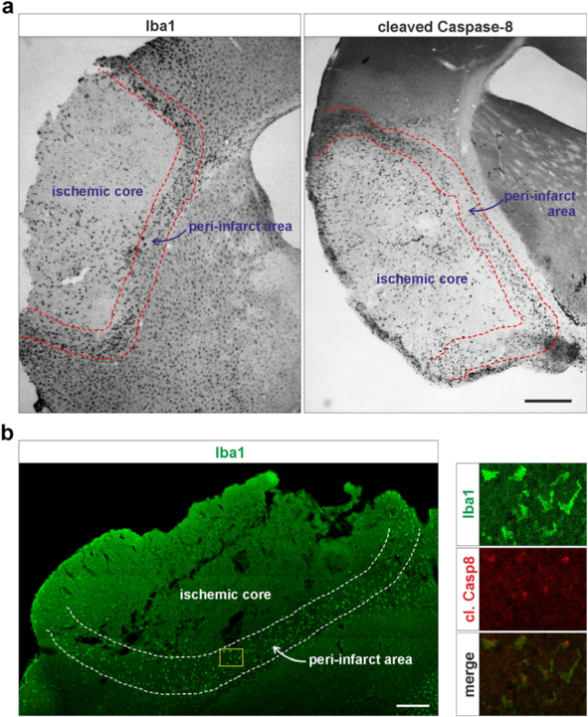
Iba1-positive myeloid cells in the ischemic peri-infarct area express cleaved caspase-8 in a pMCAO mouse model of ischemic stroke. Consecutive frozen tissue sections of the ischemic core and the peri-infarct regions from mice after 48 h of permanent middle cerebral artery occlusion (pMCAO) were analyzed by immunohistochemistry with a mouse specific antibody recognizing active caspase-8 when cleaved at Asp 387, or an Iba1 antibody used to detect myeloid cells (**a**). Double immunofluorescent staining demonstrated the co-localization of cleaved caspase-8 and the myeloid cell marker Iba1 (**b**). Scale bars represent 850 μm (**a**) and 300 μm (**b**)

Iba1 and cleaved caspase-8 positive cells were quantified after IHC staining. Analysis was performed using the 48 h post-occlusion time point on four animals. Two sections per animal were quantified and 3 fields per section were counted for each area (ischemic core, peri-infarct area and surrounding area). The peri-infarct area is defined as the region surrounding the ischemic core, approximately 500 μm wide, with a distinct activation of Iba1+ cells (illustrated in Fig. 1). The surrounding area is the region distally outside the peri-infarct area, where areas for quantification are selected approximately 1 mm outside the peri-infarct area. Cells were divided after morphological appearance as ramified, hypertrophic, amoeboid or rounded.

## Results

### Temporal and spatial activation of caspase-8 in Iba1-positive myeloid cells in a pMCAO mouse model of ischemic stroke

To investigate activation of caspase-8 in microglia/macrophages (MMs) upon a stroke event, a mouse model with a permanent middle cerebral artery occlusion (pMCAO) was used to simulate an ischemic stroke. Immunohistochemical labeling of Iba1 was used to detect the MMs present in the ischemic core, peri-infarct area and surrounding area (Fig. 1a). Active caspase-8 was detected using an antibody that recognizes the mouse caspase-8 when proteolytically activated by cleavage at amino acid residue Asp387. Positivity for cleaved caspase-8 was detected in the ischemic core and the peri-infarct area (Fig. 1a) and could be associated with the Iba1-positive cells, as illustrated with immunofluorescence double staining confirming expression of cleaved caspase-8 in Iba1-positive cells upon stroke (Fig. 1b).

**Fig. 2:**
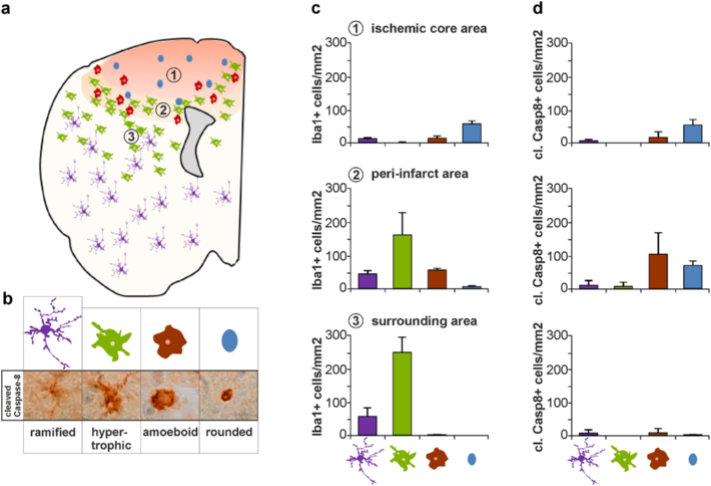
Expression of active caspase-8 correlates with the morphological transformation of Iba1-positive myeloid cells. Schematic overview of the distribution of myeloid cells in the 1) ischemic core 2) peri-infarct area and 3) surrounding area (**a**). Cell morphologies were divided into ramified, hypertrophic, amoeboid and rounded to reflect the states of activation. Examples of the different cell morphologies are given as cartoon and upon immunohistochemical staining for cleaved caspase-8 (**b**). Morphological quantifications of Iba1-positive cells (**c**) and cleaved caspase-8-positive cells (**d**) defined as cells per mm^2^ in the ischemic core (1), peri-infarct area (2) and surrounding area (3) in tissue from mice (*n* = 4) upon 48 h of pMCAO

**Fig. 3:**
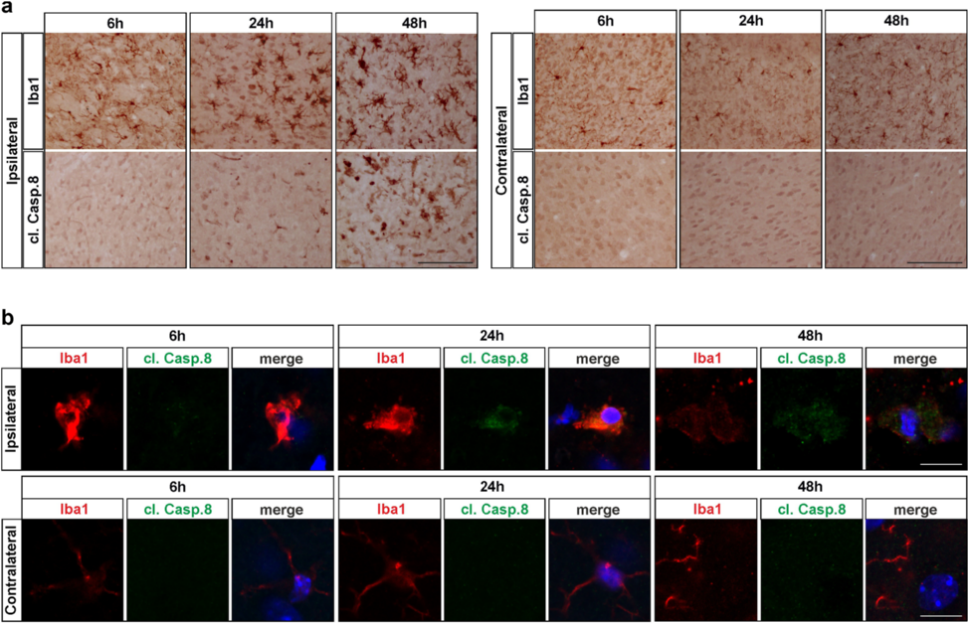
Temporal activation of caspase-8 in Iba1-positive myeloid cells. Frozen tissue sections from mice upon pMCAO (*n* = 9) were analyzed by immunohistochemistry for expression of active caspase-8 or for expression of the microglia/macrophage marker Iba1. The contralateral side to the ischemic area was used an internal control for each animal. A small increase in body size of Iba1 positive cells was detected in the peri-infarct area at 6 h, with larger increase in cell body and numbers of cells expressing Iba1 detected at 24 and 48 h post occlusion (**a**). Cleaved caspase-8 was detected at low levels at 6 h, but a significant increase in expression levels was observed at 24 and 48 h in the peri-infarct area, but not at the contralateral side (**a**). Double immunofluorescent staining revealed that increased active caspase-8 expression was localized in Iba1-positive cells (**b**). Scale bar represent 100 μm in IHC images (**a**) and 10 μm in IF images (**b**)

The brain's inflammatory response post-ischemia is characterized by several stages, which is also reflected in the recruitment and activation state of the involved immune cells. Therefore, morphological changes were analyzed in Iba1-positive cells and related to the proximity to the ischemic area. Early-stage activation is characterized by increased ramification of cytoplasmic processes and cell size and enhanced Iba1 labeling. This is followed by further thickening of processes and retraction of finer ones and increased cell body size, to end with complete retraction of cytoplasmic processes to acquire an amoeboid-shape morphology. Quantifications of Iba1-positive cells revealed highest numbers of ramified or hypertrophic cells in the surrounding area, with increasing numbers of amoeboid cells in the peri-infarct area and of rounded cells in the ischemic core, indicating higher degree of activation of cells in the proximity to the ischemic area (Fig. 2a-c). Interestingly, these morphological changes could even be visualized with the cleaved caspase-8 staining and quantified after morphological appearance. Here the largest number of cleaved caspase-8 positive cells displayed an amoeboid and rounded morphology and were present in the peri-infarct area and in the ischemic core at 48 h post occlusion (Fig. 2a-b and d). The temporal aspect of caspase-8 activation in MMs was investigated during the acute and subacute phase of inflammation, using murine brain tissue samples analyzed at 6, 24 and 48 h post artery occlusion. Morphological changes indicative of an activation of the Iba1-positive MMs were observed already at 6 h in the peri-infarct area, but were even more prominent at 24 and 48 h after occlusion (Fig. 3a). The contralateral side of the brain was used for comparison of the occurring changes in terms of cell numbers and morphological changes of the cells close to the ischemic area. Very low levels of cleaved caspase-8 could be detected at the 6 h’ time point in the peri-infarct area, but those levels were found to be significantly increased at 24 and 48 h’ time point (Fig. 3a). Double immunofluorescence staining for cleaved caspase-8/Iba1 and confocal imaging illustrated that cleaved caspase-8 was rarely detected at the 6 h occlusion, but more frequently in the cytosol of Iba1-positive cells at the 24 and 48 h time points.

### Expression of active caspase-8 and caspase-3 in CD68-positive myeloid cells in stroke subjects correlates with age of the ischemic area

**Fig. 4:**
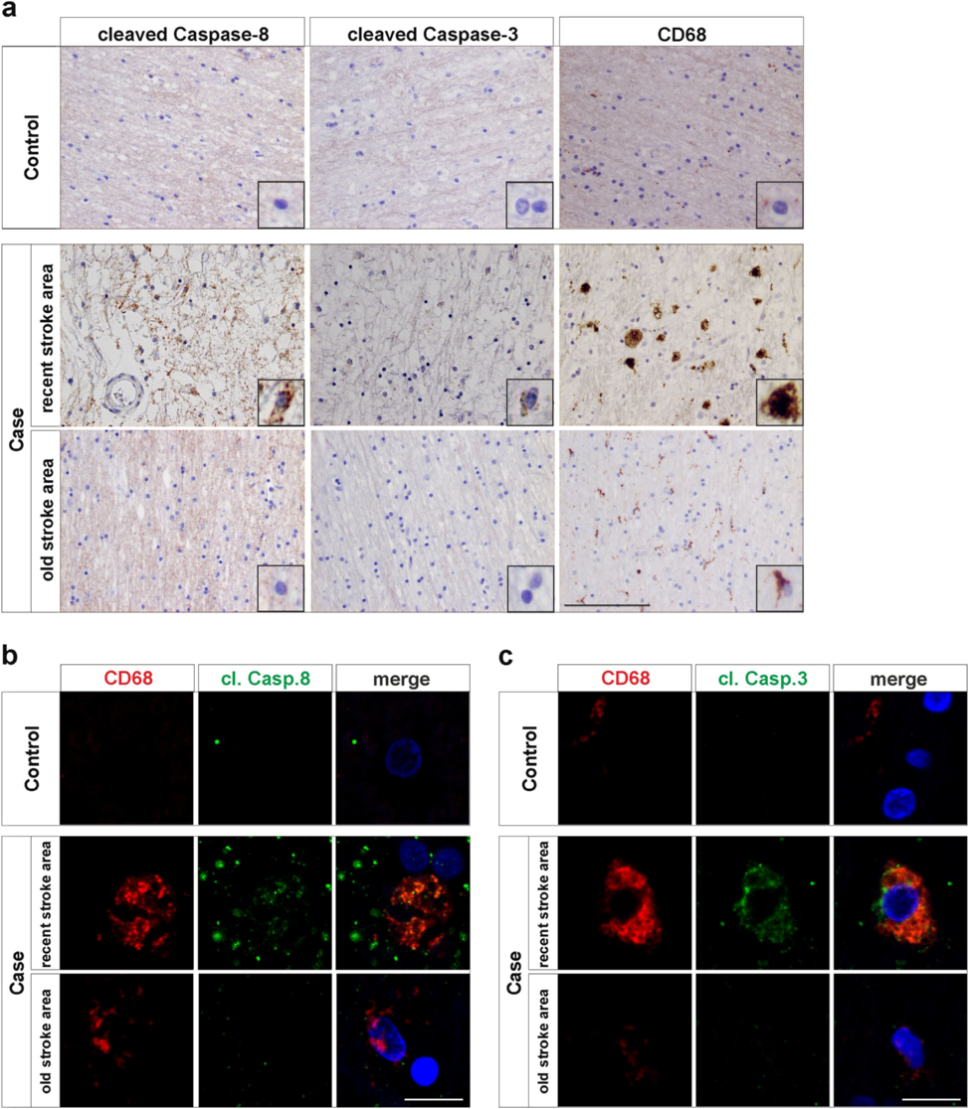
Presence of active caspase-8 and caspase-3 in CD68-positive cells correlates with age of the ischemic area. Paraffin imbedded brain tissue sections from subjects who suffered from two strokes, referred as recent and old stroke areas, were analyzed by immunohistochemistry for active caspase-8 and caspase-3 expressions using antibodies raised against caspase-8 when cleaved at Asp391 and human caspase-3 when cleaved at Asp175 and compared to healthy control tissue. Cleaved caspase-8 and -3 were detected in tissue from the recent stroke area, but not in healthy control tissue (**a**). Numbers and levels of CD68-positive cells were markedly increased in recent stroke tissue as compared to control (**a**), and found to be positive for cleaved caspase-8 and -3 using double immunofluorescence staining analyses (**b**-**c**). In contrast, for the older stroke area, lower levels of CD68 were detected as well as very low level/absence for cleaved caspase-8 and -3 (**a**). Immunofluorescence analysis confirmed the decrease/absence of active caspase-8 and -3 in the CD68-expressing cells within the older stroke area (**b**-**c**). Scale bar for IHC images corresponds to 100 μm (**a**) and for IF images 10 μm (**b**-**c**)

We were then interested to examine if activation of the apical caspase-8 and its downstream target caspase-3 could also be detected in MMs in human subjects who had suffered a stroke. Post-mortem brain tissue from subjects with a recent ischemic stroke was examined using immunohistochemical stainings. CD68 was used to detect activated MMs and revealed the presence of large numbers of inflammatory CD68-positive cells with rounded macrophage-like morphology in the peri-infarct area and ischemic core (Fig. 4a and Additional file 1a). Detection of active caspase-8 was performed using an antibody which recognizes human caspase-8 when cleaved at amino acid residue Asp391 (for antibody positive controls, see Additional file 2). Positive signal for cleaved caspase-8 was detected in numerous cells in the stroke peri-infarct area, as well as some positivity in the ischemic core (Fig. 4a and Additional file 1a). Expression of activated caspase-3 was also detected in the same areas using an antibody recognizing human caspase-3 when proteolytically activated by cleavage at amino acid residue Asp175 (Fig. 4a and Additional file 1a). In order to validate the presence of those active caspases in the CD68-positive cells, double immunofluorescent labelling for CD68 and cleaved caspase-8 or caspase-3 were performed. In fact, confocal imaging demonstrated the presence of both cleaved caspase-8 and cleaved caspase-3 in the cytoplasm of CD68-positive cells, confirming the activation for those caspases in the MMs upon stroke (Fig. 4b-c and Additional file 1b-c). Interestingly, for two stroke cases we also got the opportunity to analyze tissue samples from an older stroke area within the same cases. The subjects who had suffered two different stroke events are hereafter referred to as recent and old stroke. In contrast to the recent stroke, investigation of the older stroke area revealed low number and levels of CD68-positive cells still present around the ischemic area (Fig. 4a and Additional file 1a, compare middle “recent stroke area” with “old stroke area” rows). Expression for cleaved caspase-8 and caspase-3 was barely detected in the peri-infarct at this stage. Double staining for the markers confirm the absence/low levels of these active caspases in remaining CD68-positive cells (Fig. 4a and Additional file 1a, compare middle “recent stroke area” with “old stroke area” rows).

**Fig. 5:**
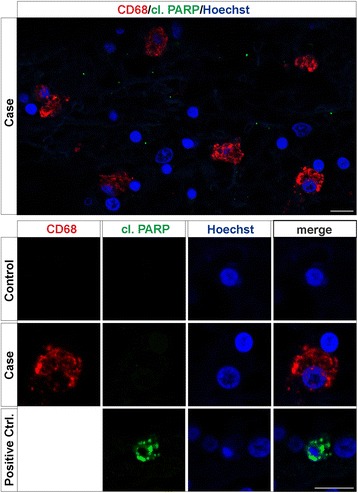
Absence of cleaved PARP, marker for apoptosis, in CD68-positive myeloid cells in stroke subject. Tissue from recent stroke case and healthy control subjected to double immunofluorescence staining using antibodies raised against CD68 and cleaved PARP, revealed an absence for the apoptosis marker in CD68-expressing myeloid cells. Tissue from colon was used as positive control for the cleaved PARP staining. Overview of the peri-infarct area of stroke case is depicted on *top panel*, whereas higher magnification images for both stroke case and controls are presented in the *lower panels*. Scale bars in images represent 10 μm

Under cell death conditions, activated caspase-8 is known to cleave and activate caspase-3, which in turn can cleave PARP and contribute to the demise of the cell. To demonstrate absence of a caspase-dependent cell death in the CD68-positive myeloid cells, tissue from control subjects and recent stroke areas in patients were labelled with antibodies detecting cleaved PARP and CD68. We could not detect PARP cleavage in CD68 positive cells either in the ischemic core or peri-infarct area after stroke, indicating that the caspase activation in these cells is not linked to cell death (Fig. 5). PARP cleavage was, however, detected in the colon tissue used as positive control (Fig. 5).

In a panel of 9 subjects who had suffered a stroke, post mortem tissue from 10 different stroke areas of various ages (i.e. time after stroke onset) were examined. Evaluation of the tissue by hematoxylin and eosin staining was used to detect and determine the age of the stroke areas (not included in manuscript). In addition, five area and age-matched controls were also included in the investigation. Immunohistochemical staining for CD68, active caspase-8 and active caspase-3 was performed as described above. Scoring of the tissue for expression of the different markers was done by an experienced neuropathologist. The following scale was used for scoring: not present (-), present (+) or high levels (++) of active caspases. In the human stroke subjects, highest levels of active caspase-8 and active caspase-3 could be detected the first day after the stroke, and then slowly decreases with time until complete disappearance after 5–29 days after the ischemic event (Table 1). CD68-positivity was reported as upregulated (Yes) or basal/very low levels (No). The high numbers of CD68-positive cells were, as the caspases, detected at the earlier time points after the ischemic event, but declined with time and are back to basal levels (as compared to controls) in most subjects after 5–29 days after onset (Table 1).

**Table 1:** Temporal expression of active caspase-8 and caspase-3 in a panel of stroke subjects

	Cleaved Caspase-8	Cleaved Caspase-3	CD68 upregulated	Days after stroke
Case 1	++	++	Yes	0–1
Case 2	++	+	Yes	0–1
Case 3	+	+	Yes	0–1
Case 4	+	+	Yes	1–4
Case 5	+	–	Yes	1–4
Case 6	+	–	Yes	5–29
Case 7a	+	–	No	5–29
Case 8	+	–	No	5–29
Case 7b	–	–	No	30–
Case 9	–	–	No	30–
Control 1	–	–	No	
Control 2	–	–	No	
Control 3	–	–	No	
Control 4	–	–	No	
Control 5	–	–	No	

Tissue from 10 different areas from stroke subjects (Case; *n* = 9) and 5 areas from healthy controls (*n* = 5) were analyzed for CD68, cleaved caspase-8 and cleaved caspase-3 expression by immunohistochemistry (exemplified in Fig. 4a). Cleaved caspase-8 or -3 expression levels were scored as (-) not present, (+) present or (++) high levels. CD68 expression was represented as (No) not present/basal levels or (Yes) increase in CD68 positive cells. Age of stroke area was determined by hematoxylin and eosin staining and is presented as days after stroke event. Highest levels of cleaved caspase-8 and-3 expressions were found within the first days after stroke. They were found to decrease with time, and were completely gone within 30 days. CD68-positive cells can be found at high numbers within the first days after stroke and decrease to basal levels within 30 days after stroke onset

## Discussion

There is compelling evidence that brain injury following ischemic stroke develops from a complex series of pathophysiological events that evolve in time and space [19, 20]. After an ischemic stroke, experimental and clinical data suggest that a prominent inflammatory response develops, propagates, and lasts for many days, and is believed to exacerbate neuronal cell death [21, 22].

The brain’s initial inflammatory response to stroke is proposed to be primarily mediated by microglia, the resident immune cells of the CNS. However, within minutes or hours of the stroke event, the blood–brain barrier is compromised and infiltration of monocytes, neutrophils and lymphocytes occurs [23, 4, 24]. The brain-resident (microglia) and infiltrating peripheral (monocytes) myeloid cells have a prominent role in initiating, sustaining and resolving post-ischemic inflammation. It is therefore of importance to elucidate the molecular mechanism regulating their activation. Our team previously described an unexpected novel function for caspases in the control of microglia activation and thereby neurotoxicity. We showed that orderly activation of caspase-8 and caspase-3 regulates microglia activation, in the absence of cell death [11]. In addition, we recently obtained evidence that caspase-8 regulates the activation of human monocytes [12]. Considering the central role played by these caspases in the activation of microglia/monocytes, and the contribution of these cells in the observed inflammatory response following ischemic stroke, we decided to investigate whether activation of these caspases follow spatial and temporal features.

Immunohistochemical staining, as well as immunofluorescence confocal imaging, of post-mortem tissues from subjects who had suffered an ischemic stroke, was used with a CD68-antibody to detect activated myeloid cells. Additional staining with cleaved caspase-8 or cleaved caspase-3 revealed that myeloid cells in the ischemic core and peri-infarct area expressed active caspase-8 and caspase-3. It is believed that non-apoptotic functions of caspases rely on a moderate activity and a restricted subcellular localization. We have demonstrated that a differential processing of caspase-3 zymogen may ultimately lead to apoptosis (caspase-3 subunit p17; nuclear localization) or microglia activation (caspase-3 subunit p19; cytosolic localization) [25]. Our confocal analysis demonstrated a non-nuclear localization of active caspase-3 within myeloid cells early after stroke, a view that fits well with the non-apoptotic role of caspases in regulating myeloid cell activation. Analysis of brain tissue samples from a pMCAO mouse model of ischemic stroke, at 6, 24 and 48 h post artery occlusion, illustrated a temporal and spatial activation for caspase-8 in Iba1-positive myeloid cells. Indeed, increased levels for cleaved caspase-8 staining were found to correlate with morphological changes of the Iba1-positive cells from ramified cells to amoeboid or rounded shapes in proximity to the ischemic core. Notably, this correlation was particularly evident in the peri-infarct area, a region revealing penumbra like conditions and is potentially salvable upon a brain infarct, in contrast to the stroke core where perfusion is completely absent and irreversible loss of tissue (infarction) occurs within minutes [26]. It has been long established that microglia activation is particularly evident in the penumbra region in response to ischemic damage [19]. Although the contribution of the inflammatory response to ischemic brain injury is under debate, increasing evidence points out a deleterious role. For instance, recent data have demonstrated how microglia in the penumbra region is strongly associated with blood vessels; reactive microglia phagocytose endothelial cells, thus contributing to BBB disruption and neurodegeneration [27]. In the present study we demonstrate the potential contribution of caspases in regulating brain immune function in the peri-infarct penumbra-like region. The multifaceted roles of caspase-8 make this caspase very attractive in the context of future stroke strategies aimed at minimizing brain injury. These varied roles include i) it controls microglia activation through a caspase-3/NF-kB-dependent mechanism, with the subsequent release of neurotoxic proinflammatory cytokines [11], ii) recent data suggest a prominent role of this caspase in the priming and activation of inflammasomes [13], iii) inflammasome activation may lead to pyroptosis, a proinflammatory and lytic mode of cell death and iiii) caspase-8 negatively regulates programmed necrosis or necroptosis, which relies of a molecular platform known as necrosome and strongly associated to immune cells of myeloid origin including microglia, monocytes and macrophages [28]. Since the repair process for damaged brain tissues and regeneration of neural cells takes place during resolution of inflammation [17], and having demonstrated that active caspase-8 is mostly confined to reactive myeloid cells in the peri-infarct region, we anticipate that site-directed delivery of caspase-8 inhibitors under stroke conditions may hinder microglia activation and affect microglia survival. Finally, our analysis of post-mortem tissues from subjects who suffered two independent ischemic stroke events, as well as the examination of panel of 10 different stroke areas of various ages (i.e. time after stroke onset) suggested that the presence of active caspase-8 and -3 in CD68-positive cells correlates with the age of ischemic area.

The approximate time after stroke onset can be determined by neuropathologists and forensic pathologists by the postmortem analysis of ischemic lesions. Parameters used may include analysis of inflammation and myeloid component [29, 30]. The distinct pattern of cleaved caspase-8 surrounding the infarct in our data suggests that immunohistochemical analysis of cleaved caspase-8 could be considered as a relevant additional diagnostic parameter.

Whereas inhibition of caspase-8 and/or caspase-3 could be used as therapeutic strategy to combat the inflammatory response initiated upon ischemic stroke remains controversial. Evidence for neuroprotection have been reported with Quinolyl-valyl-O-methylaspartyl-[-2,6-difluorophenoxy]-methylketone (Q-VD-OPh) a third-generation broad spectrum caspase inhibitor [31], which is able to crosses the blood–brain barrier, in animal models of stroke [32]. However, it remains to be established whether Q-VD-OPh affects microglia/monocytes activation in this disease model and contributes to the neuroprotective effect in that manner. In addition, Q-VD-OPh treatment has been reported to impair the neural stem/progenitor cell response after cortical ischemia in mice, highlighting that caution is warranted using such strategy [33].

## Conclusion

In conclusion, we revealed the temporal and spatial activation of caspase-8 and -3 in microglia/macrophages occurring upon ischemic stroke in human stroke subjects and in a mouse model of ischemic stroke. Overall, the report of a spatio-temporal activation of caspase-8 and -3 in microglia/macrophages occurring upon ischemic stroke indicates that any attempt to target the molecular signaling regulating the detrimental inflammatory response would have to take into account the time window for intervention.

## Additional files

Additional file 1: Figure S1. Analysis of a second subject whom suffered from two stroke events and a healthy control. Immunohistochemical labelling for CD68, cleaved caspase-8 and cleaved caspase-3 (a), as well as double immunofluorescent labelling for CD68 and cleaved caspase-8 alternatively cleaved caspase-3 (b-c) in recent and old stroke areas are depicted. IHC image scale bar corresponds to 100 μm (a) and IF images to 10 μm (b-c). (TIF 4099 kb) Additional file 2: Figure S2. Positive controls for immunohistochemistry antibodies. Antibodies detecting cleaved caspase-8 and cleaved caspase-3 showed positive signal in sections from human colon tissue. Antibody detecting CD68 showed positive signal in sections from human lymph node. Scale bar represents 100 μm. (TIF 1191 kb)

## Abbreviations

CNS: Central nervous system
DAMPs: Danger-associated molecular patterns
HE: Hematoxylin-eosin
IF: Immunofluorescence
IHC: Immunohistochemistry
LFB: Luxol fast blue/cresyl violet
MM: Microglia/macrophages
pMCAO: Permanent middle cerebral artery occlusion
Q-VD-OPh: Quinolyl-valyl-O-methylaspartyl-[-2,6-difluorophenoxy]-methylketone

## References

[1] Feigin VL, Forouzanfar MH, Krishnamurthi R, Mensah GA, Connor M, Bennett DA, Moran AE, Sacco RL, Anderson L, Truelsen T, O'Donnell M, Venketasubramanian N, Barker-Collo S, Lawes CM, Wang W, Shinohara Y, Witt E, Ezzati M, Naghavi M, Murray C, Global Burden of Diseases Ij, and Risk Factors Study 2010 (GBD 2010) and the GBD Stroke Experts Group (2014). Global and regional burden of stroke during 1990–2010: findings from the Global Burden of Disease Study 2010. Lancet.

[2] Collaborators GBoDS (2015). Global, regional, and national incidence, prevalence, and years lived with disability for 301 acute and chronic diseases and injuries in 188 countries, 1990–2013: a systematic analysis for the Global Burden of Disease Study 2013. Lancet.

[3] Donnan GA, Fisher M, Macleod M, Davis SM (2008). Stroke. Lancet.

[4] Iadecola C, Anrather J (2011). The immunology of stroke: from mechanisms to translation. Nat Med.

[5] Kreutzberg GW (1996). Microglia: a sensor for pathological events in the CNS. Trends Neurosci.

[6] Jin R, Yang G, Li G (2010). Inflammatory mechanisms in ischemic stroke: role of inflammatory cells. J Leukoc Biol.

[7] Weinstein JR, Koerner IP, Möller T (2010). Microglia in ischemic brain injury. Future Neurol.

[8] Nilupul Perera M, Ma HK, Arakawa S, Howells DW, Markus R, Rowe CC, Donnan GA (2006). Inflammation following stroke. J Clin Neurosci.

[9] Tuttolomondo A, Pecoraro R, Arnao V, Maugeri R, Iacopino DG, Pinto A (2015). Developing drug strategies for the neuroprotective treatment of acute ischemic stroke. Expert Rev Neurother.

[10] Venero JL, Burguillos MA, Joseph B (2013). Caspases playing in the field of neuroinflammation: old and new players. Dev Neurosci.

[11] Burguillos MA, Deierborg T, Kavanagh E, Persson A, Hajji N, Garcia-Quintanilla A, Cano J, Brundin P, Englund E, Venero JL, Joseph B (2011). Caspase signalling controls microglia activation and neurotoxicity. Nature.

[12] Oliva-Martin MJ, Sanchez-Abarca LI, Rodhe J, Carrillo-Jimenez A, Vlachos P, Herrera AJ, Garcia-Quintanilla A, Caballero-Velazquez T, Perez-Simon JA, Joseph B, Venero JL (2016). Caspase-8 inhibition represses initial human monocyte activation in septic shock model.

[13] Gurung P, Kanneganti TD (2015). Novel roles for caspase-8 in IL-1β and inflammasome regulation. Am J Pathol.

[14] Man SM, Kanneganti TD (2015). Regulation of inflammasome activation. Immunol Rev.

[15] Kavanagh E, Burguillos MA, Carrillo-Jimenez A, Oliva-Martin MJ, Santiago M, Rodhe J, Joseph B, Venero JL (2015). Deletion of caspase-8 in mouse myeloid cells blocks microglia pro-inflammatory activation and confers protection in MPTP neurodegeneration model. Aging (Albany NY).

[16] Gurung P, Anand PK, Malireddi RK, Vande Walle L, Van Opdenbosch N, Dillon CP, Weinlich R, Green DR, Lamkanfi M, Kanneganti TD (2014). FADD and caspase-8 mediate priming and activation of the canonical and noncanonical Nlrp3 inflammasomes. J Immunol.

[17] Shichita T, Ito M, Yoshimura A (2014). Post-ischemic inflammation regulates neural damage and protection. Front Cell Neurosci.

[18] Lambertsen KL, Clausen BH, Babcock AA, Gregersen R, Fenger C, Nielsen HH, Haugaard LS, Wirenfeldt M, Nielsen M, Dagnaes-Hansen F, Bluethmann H, Faergeman NJ, Meldgaard M, Deierborg T, Finsen B (2009). Microglia protect neurons against ischemia by synthesis of tumor necrosis factor. J Neurosci.

[19] Dirnagl U, Iadecola C, Moskowitz MA (1999). Pathobiology of ischaemic stroke: an integrated view. Trends Neurosci.

[20] Moskowitz MA, Lo EH, Iadecola C (2010). The science of stroke: mechanisms in search of treatments. Neuron.

[21] Wang Q, Tang XN, Yenari MA (2007). The inflammatory response in stroke. J Neuroimmunol.

[22] Ceulemans AG, Zgavc T, Kooijman R, Hachimi-Idrissi S, Sarre S, Michotte Y (2010). The dual role of the neuroinflammatory response after ischemic stroke: modulatory effects of hypothermia. J Neuroinflammation.

[23] Ritzel RM, Patel AR, Grenier JM, Crapser J, Verma R, Jellison ER, McCullough LD (2015). Functional differences between microglia and monocytes after ischemic stroke. J Neuroinflammation.

[24] Benakis C, Garcia-Bonilla L, Iadecola C, Anrather J (2014). The role of microglia and myeloid immune cells in acute cerebral ischemia. Front Cell Neurosci.

[25] Kavanagh E, Rodhe J, Burguillos MA, Venero JL, Joseph B (2014). Regulation of caspase-3 processing by cIAP2 controls the switch between pro-inflammatory activation and cell death in microglia. Cell Death Dis.

[26] Dwyer TA, Earl DE, Wang L (2008). The utility of a new in vitro model of the stroke penumbra. J Neurosci.

[27] Jolivel V, Bicker F, Binamé F, Ploen R, Keller S, Gollan R, Jurek B, Birkenstock J, Poisa-Beiro L, Bruttger J, Opitz V, Thal SC, Waisman A, Bäuerle T, Schäfer MK, Zipp F, Schmidt MH (2015). Perivascular microglia promote blood vessel disintegration in the ischemic penumbra. Acta Neuropathol.

[28] Pasparakis M, Vandenabeele P (2015). Necroptosis and its role in inflammation. Nature.

[29] Love S (2011). Autopsy approach to stroke. Histopathology.

[30] Mena H, Cadavid D, Rushing EJ (2004). Human cerebral infarct: a proposed histopathologic classification based on 137 cases. Acta Neuropathol.

[31] Chauvier D, Ankri S, Charriaut-Marlangue C, Casimir R, Jacotot E (2007). Broad-spectrum caspase inhibitors: from myth to reality?. Cell Death Differ.

[32] Braun JS, Prass K, Dirnagl U, Meisel A, Meisel C (2007). Protection from brain damage and bacterial infection in murine stroke by the novel caspase-inhibitor Q-VD-OPH. Exp Neurol.

[33] Osman AM, Neumann S, Kuhn HG, Blomgren K (2015). Caspase inhibition impaired the neural stem/progenitor cell response after cortical ischemia in mice.

